# Does acute kidney injury alerting improve patient outcomes?

**DOI:** 10.1186/s12882-022-03031-y

**Published:** 2023-01-17

**Authors:** Jolene Atia, Felicity Evison, Suzy Gallier, Peter Hewins, Simon Ball, Joseph Gavin, Jamie Coleman, Mark Garrick, Tanya Pankhurst

**Affiliations:** 1 University Hospitals Birmingham NHS Foundation Trust, Edgbaston, Birmingham, UK; 2 University Hospitals Birmingham NHS Foundation Trust, Institute of Translational Medicine, Edgbaston, Birmingham, UK; 3 PIONEER: HDR-UK Health Data Research Hub for Acute Care, University of Birmingham, Birmingham, UK; 4 HDRUK Better Care Science Priority and Health Data Research UK Midlands, University of Birmingham, Birmingham, UK; 5 Lister Hospital, Stevenage, UK; 6 School of Medicine, College of Medical and Dental Sciences, University of Birmingham, Edgbaston, Birmingham, UK; 7 Queen Elizabeth Hospital Birmingham, Mindelsohn Way, Birmingham, B15 2TH UK

**Keywords:** Acute Kidney Injury, Patient outcomes, Referral, Electronic Health records, Electronic Patient Records

## Abstract

**Background:**

Electronic alerts (e-alerts) for Acute Kidney Injury (AKI) have been implemented into a variety of different Electronic Health Records (EHR) systems worldwide in order to improve recognition and encourage early appropriate management of AKI. We were interested in the impact on patient safety, specialist referral and clinical management.

**Methods:**

All patients admitted to our institution with AKI were included in the study. We studied AKI progression, dialysis dependency, length of hospital stay, emergency readmission, ICU readmission, and death, before and after the introduction of electronic alerts. The impact on prescription of high risk drugs, fluid administration, and referral to renal services was also analysed.

**Results:**

After the introduction of the e-alert, progression to higher AKI stage, emergency readmission to hospital and death during admission were significantly reduced. More prescriptions were stopped for drugs that adversely affect renal function in AKI and there was a significant increase in the ICU admissions and in the number of patients having dialysis, especially in earlier stages. Longer term mortality, renal referrals, and fluid alteration did not change significantly after the AKI e-alert introduction.

**Conclusions:**

AKI e-alerts can improve clinical outcomes in hospitalised patients.

**Supplementary Information:**

The online version contains supplementary material available at 10.1186/s12882-022-03031-y.

## Key learning points

### Evidence before this study

- We searched for articles published in English using the search terms “Acute Kidney Injury”, “AKI”, “e-Alerts”, “Clinical Decision Support” for studies on electronic clinical decision support (CDS) in different medical settings. We also used the NICE guidelines and references for definitions of AKI using KDIGO classification.
- There has been conflicting opinion regarding the effect of AKI CDS in improving patient’s outcomes.

### Added value of this study

- This is an analysis to study the effect of AKI CDS tool if implemented within a locally developed Electronic Health Record (EHR).
- We used an extensive data cohort available from the EHR in a large hospital to study the cohorts two years before and two years after the AKI alerts were introduced.
- We looked at patient-related, processes of care and health outcomes.

### Impact of all the available evidence

In a well-designed, fully integrated in EHR, AKI alerting can positively impact patient outcome.

## Introduction

Acute kidney injury (AKI) is one of the most serious and common complications affecting medical inpatient admissions and is associated with high mortality [1]. Early detection and appropriate management of AKI is vital to aid recovery, prevent further kidney injury [2, 3] and provide appropriate on-going supportive management [3]. Initial recommended management of AKI is focused on correcting fluid and electrolyte imbalance, discontinuation or avoidance of nephrotoxins, drug dose adjustments, and if necessary admission to intensive care or referral to renal services [3, 4]. There is evidence to suggest that deficiencies in clinical care may contribute to the progression of the condition [5]. A report by the National Confidence Enquiry into Patient Outcome and Death (NCEPOD) found that 30% of AKI cases occurring during hospital admission were avoidable and that only 50% of patients with AKI received the appropriate standard of care [6].

To improve recognition of AKI, electronic alerts were recommended and an e-alert system for AKI was mandated by NHS England in all Laboratory Information Management Systems, across the NHS in 2015 [2, 7]. National electronic alerts are based on an algorithm described in the NICE guidelines NG148 [8], which defines AKI using the Kidney Disease: Improving Global Outcomes (KDIGO) classification [9], which was commonly used amongst other studies [10–14].

Since the national implementation of AKI alerts in UK several studies have endeavoured to understand their impact. Five found that the e-alerts improved outcomes [11, 12, 15–18], while other studies found no or modest effect [2, 13, 19, 20].

The overall aim of this study was to assess the effect of an AKI Clinical Decision Support (CDS) tool implemented within a locally developed electronic health record (EHR) system and whether it has any effect on the quality of care and patient outcomes.

## Methods

### Setting and context

This study was conducted in a large, urban teaching hospital within the UK, that has an in-house built, clinically-led Electronic Health Record (EHR); PICS (Prescribing, Information and Communication System) which has been described elsewhere [21, 22]. PICS provides users with CDS, based on user privilege, clinical protocols and best practice guidelines. Alerts and advice appear to the prescriber as orders. These are tiered in severity and can be interruptive or non-interruptive [23].

The hospital has the largest Critical Care Unit in Europe, with up to 100 bed spaces; it is a centre for major trauma and a tertiary referral centre for many specialities including oncology and nephrology.

### Description of the intervention

The national AKI alert in the UK was introduced in March 2015. In our hospital, the laboratory administration system automatically calculates the AKI stage, according to KDIGO guidelines whenever a new creatinine result becomes available. The baseline creatinine used is either the lowest creatinine result in the last 7 days or the median of all the creatinine results in the last 365 days. A significant rise of the serum creatinine would categorise the AKI stage [8].

**Fig. 1:**
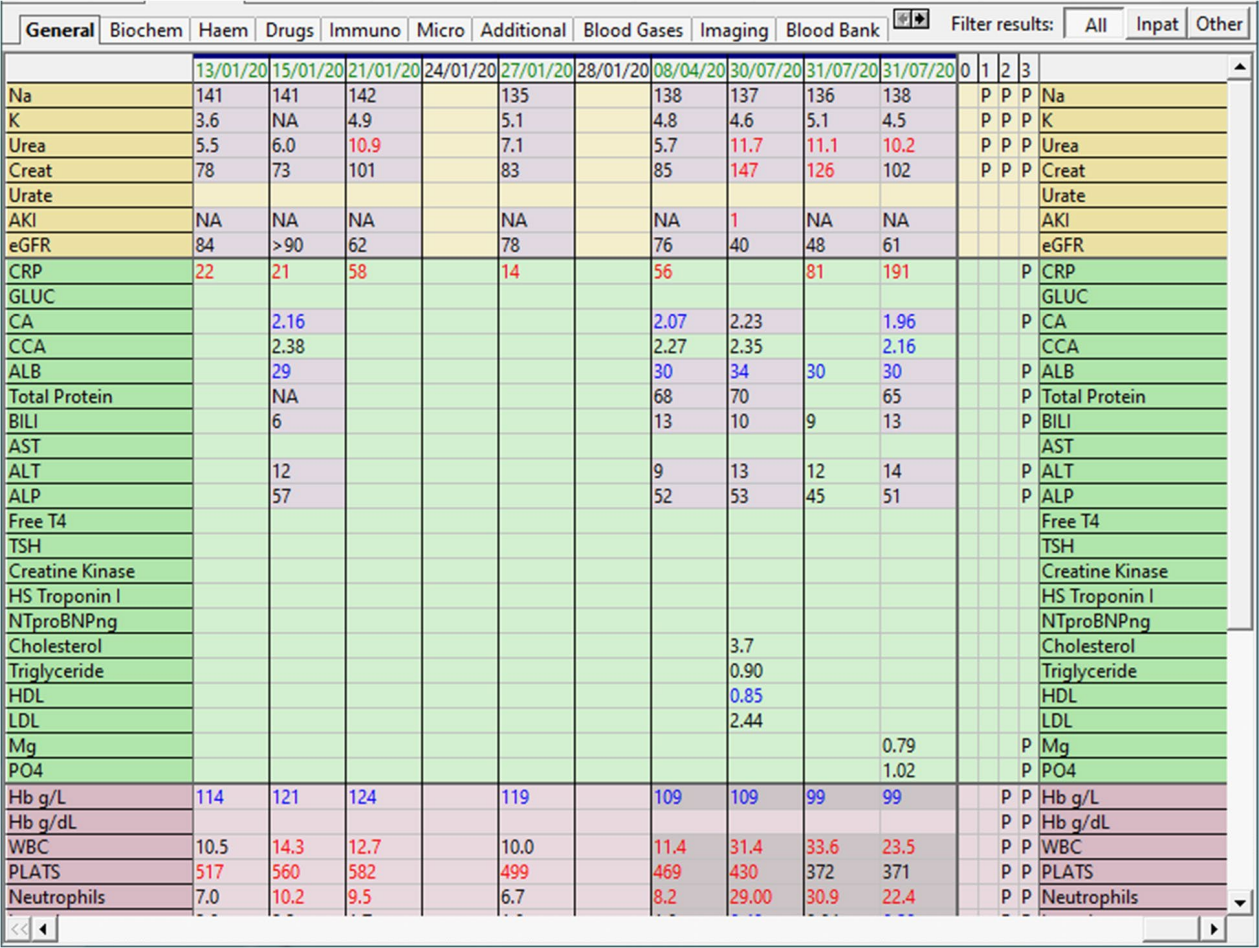
AKI e-alert based on delta change in creatinine (figure reproduced in black and white in printed version; coloured version available online in the supplementary material)

**Fig. 2:**
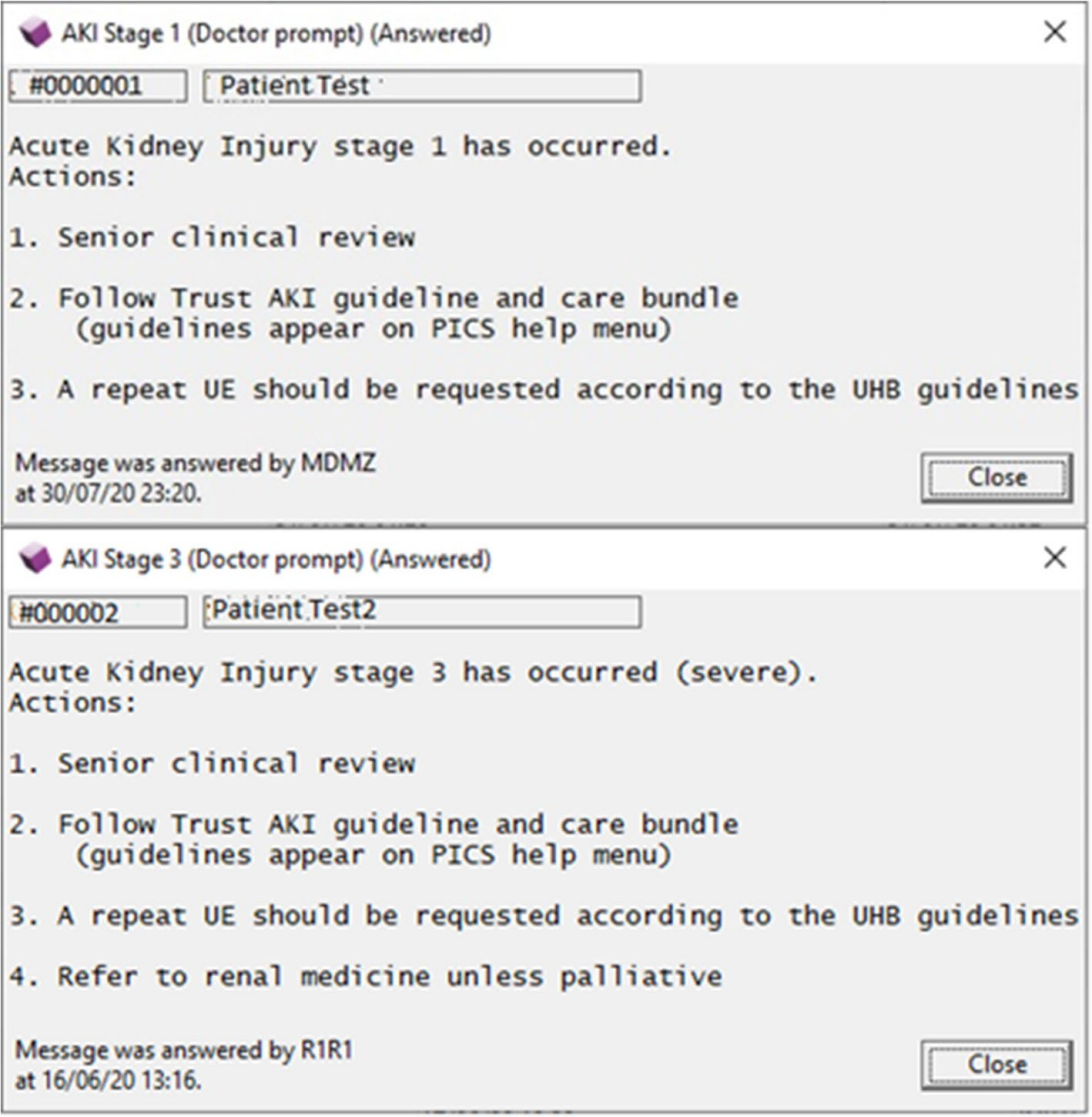
AKI doctor prompts (AKI stage 1 and 3)

AKI stage is sent as a result into the EHR and is displayed in red in the results flowsheets (Fig. 1) alongside the creatinine. In addition, an interruptive alert appears to clinicians advising of the AKI stage and the level of severity (Fig. 2).

### Study population and data extraction

Two epochs were studied; a pre-intervention epoch (epoch 1) before AKI alerts were mandated nationally, and a post-intervention epoch (epoch2) after the introduction of the alert. For epoch 1, patient-level data on all adult inpatients, who would have triggered an AKI alert according to the KDIGO guidelines between 15^th^ April 2013 and 14^th^ April 2015 were extracted from the EHR. For the epoch 2, data on all adult inpatients who triggered the AKI alert between 15^th^ April 2015 and 14^th^ April 2017 was collected. Data collection was restricted to the first creatinine that triggered the AKI alert (or would have triggered in the pre alert population) to prevent multiplication of data and consequent bias in the population.

**Fig. 3:**
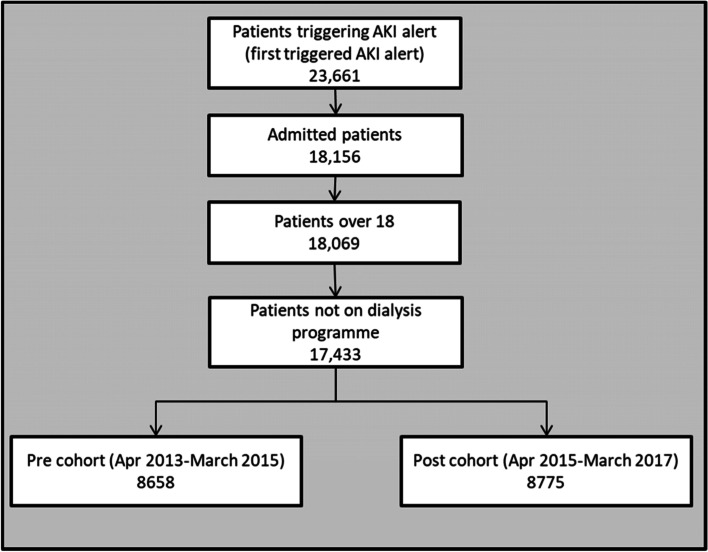
Patient recruitment flow chart

For each patient we extracted the age, sex, ethnicity, and weight and height (for BMI calculation), which were categorised as detailed in Table 1. CRP (C-reactive protein-the most used inflammatory marker in the UK) on admission was extracted and separated into three groups (normal: ≤ 5 mg/L, high: > 5 mg/L, unknown), and creatinine on admission into three groups (low: <  = 90, high: > 90, unknown). A modified (excluding renal disease) Charlson comorbidity score, previously validated for use in the NHS [24, 25], was calculated, using all of the patient’s pre-existing comorbidities, and categorised into three groups (no records or 0, 1–4, ≥ 5). Renal comorbidity was used in conjunction with high creatinine on admission to create a binary variable as a proxy for an existing renal problem. We also extracted the first AKI stage of each patient, the next AKI stage recorded, the date of any dialysis procedures, commonly used nephrotoxic drugs and commonly administered fluids prescribed to the patient (see supplementary material), the admission and discharge dates, any subsequent emergency readmissions, any following referrals to renal during admission or ICU within 24 h, and the date of death where relevant. Patients who were already on a dialysis programme were excluded (Fig. 3).

**Table 1:** Patients characteristics

**Baseline patients characteristics**				**Matched patients characteristics**		
				(caliper = 0.04-Matched all except age including First AKI)		
	Pre-cohort	Post-cohort	*p* value	Pre-cohort	Post-cohort	*p* value
**Number of patients**	8775 (50.3%)	8658 (49.7%)		7783	7783	
**Age**			0.0537			0.279
200318–34	654 (7.6%)	633 (7.2%)		592 (7.6%)	594 (7.6%)	
35–44	587 (6.8%)	560 (6.4%)		522 (6.7%)	511 (6.6%)	
45–54	1083 (12.5%)	1076 (12.3%)		979 (12.6%)	973 (12.5%)	
55–64	1509 (17.4%)	1412 (16.1%)		1367 (17.6%)	1263 (16.2%)	
65–74	1790 (20.7%)	1909 (21.8%)		1636 (21%)	1711 (22%)	
75 and above	3035 (35%)	3185 (36.3%)		2687 (34.5%)	2731 (35.1%)	
**First AKI**			< 0.001			0.068
Stage 1	6936 (80.1%)	7463 (85.1%)		6515 (83.7%)	6497 (83.5%)	
Stage 2	954 (11%)	872 (9.9%)		784 (10.1%)	851 (10.9%)	
Stage 3	768 (8.9%)	440 (5%)		484 (6.2%)	435 (5.6%)	
**Sex**			< 0.001			0.748
Male	4461 (51.5%)	4820 (54.9%)		4070 (52.3%)	4049 (52%)	
Female	4197 (48.5%)	3955 (45.1%)		3713 (47.7%)	3734 (48%)	
**Ethnicity**			< 0.001			0.732
White	7166 (82.8%)	6989 (79.7%)		6437 (82.7%)	6474 (83.2%)	
Non-white	1407 (16.3%)	1499 (17.1%)		1264 (16.2%)	1230 (15.8%)	
Unknown	85 (1%)	287 (3.3%)		82 (1.1%)	79 (1%)	
**BMI**			< 0.001			0.065
Underweight (< 18.5)	593 (6.9%)	463 (5.3%)		476 (6.1%)	452 (5.8%)	
Normal (18.5–24.9)	3010 (34.8%)	2973 (33.9%)		2718 (34.9%)	2613 (33.6%)	
Overweight (25–30)	2513 (29%)	2707 (30.9%)		2373 (30.5%)	2338 (30%)	
Obese (> 30)	2007 (23.2%)	2230 (25.4%)		1863 (23.9%)	1996 (25.7%)	
Unknown	535 (6.2%)	402 (4.6%)		353 (4.5%)	384 (4.9%)	
**CRP on admission**			< 0.001			0.222
Low (≤ 5 mg/L)	1789 (20.7%)	2050 (23.4%)		1692 (21.7%)	1662 (21.4%)	
High (> 5 mg/L)	4281 (49.5%)	4170 (47.5%)		3817 (49%)	3748 (48.2%)	
Unknown	2588 (29.9%)	2555 (29.1%)		2274 (29.2%)	2373 (30.5%)	
**Pre-existing renal problem**			< 0.001			0.176
No	4075 (47.1%)	3704 (42.2%)		3638 (46.7%)	3555 (45.7%)	
Yes	3525 (40.7%)	3988 (45.5%)		3169 (40.7%)	3181 (40.9%)	
Unknown	1058 (12.2%)	1083 (12.3%)		976 (12.5%)	1047 (13.5%)	
**Charlson score**			< 0.001			0.996
0	2902 (33.5%)	2685 (30.6%)		2443 (31.4%)	2448 (31.5%)	
01-Apr	1237 (14.3%)	1253 (14.3%)		1084 (13.9%)	1083 (13.9%)	
≥ 5	4519 (52.2%)	4837 (55.1%)		4256 (54.7%)	4252 (54.6%)	
**Progression to Higher AKI within 7 days of alert**	1413 (17.9%)	1228 (14.7%)	< 0.001	1343 (18.4%)	1089 (14.8%)	< 0.001
**Drug stopped within 12 h of alert**	228 (2.6%)	324 (3.7%)	< 0.001	214 (2.8%)	281 (3.6%)	0.0026
**Fluid altered within 12 h of alert**	1522 (17.6%)	1539 (17.5%)	0.9596	1395 (17.9%)	1364 (17.5%)	0.5289
**ICU referral within 24 h of alert**	84 (1%)	122 (1.4%)	0.0125	69 (0.9%)	108 (1.4%)	0.0041
**Renal referral during admission**	632 (7.3%)	721 (8.2%)	0.0255	558 (7.2%)	621 (8%)	0.0604
**Emergency readmission within 30 days of discharge**	1207 (16.5%)	1103 (14.7%)	0.0026	1069 (16.3%)	968 (14.5%)	0.0055
**Dialysis within 30 days after the alert**	336 (3.9%)	376 (4.3%)	< 0.001	262 (3.4%)	340 (4.4%)	< 0.001
**Death during admission**	1337 (15.2%)	1262 (14.6%)	0.0518	1203 (15.5%)	1103 (14.2%)	0.0255
**Death within 90 days**	2114 (24.4%)	2061 (23.5%)	0.1556	1900 (24.4%)	1831 (23.5%)	0.2017
**Death within a year**	2990 (34.5%)	2934 (33.4%)	0.1297	2698 (34.7%)	2618 (33.6%)	0.1818
**LOS**	15 (IQR 7–29)	14 (IQR 7–28)	0.009	16 (IQR 8–30)	15 (IQR 8–28)	0.088

Univariable analysis comparing pre and post cohort and *p* values. Values are number of patients with percentage in parentheses

### Data analysis

To analyse the effect of introducing the AKI alert we looked at a number of patient-related, process of care and health outcomes, following the first AKI: (i) progression to a higher AKI stage within seven days, (ii) having dialysis within 30 days, (iii) having nephrotoxic drugs (angiotensin converting enzyme inhibitors (ACEi) or Angiotensin Receptor Blockers (ARBs), Furosemide, non-steroidal anti-inflammatory drugs (NSAIDs), gentamicin, and/or amphotericin) stopped within 12 h, (iv) having fluids stopped or started within 12 h, (v) length of hospital stay in days (LOS) for the index admission, (vi) emergency readmission within 30 days of the discharge from the index admission (vii) renal referral during the index admission, (viii) unplanned ICU admission within 24 h, (ix) death within 90 days (x)death within a year, (xi) and death during index admission. We looked at all cause deaths, and not only renal related deaths.

Data was analysed in R (R Core team, 2020-Rsudio Version 1.1.442). The Chi-square test was used to compare the different patient characteristics and to compare the categorical variables in epoch 1 and 2 (Table 1). Patients who died during the index admission were excluded from the emergency readmission within 30 days of discharge analysis. Patients who had AKI stage 3 as their first AKI stage were excluded from the progression to the next stage analysis. Wilcoxon test was used to test the differences between the medians of LOS in both groups. Kaplan–Meier graphs of the data were constructed for the long term variables. The curves of the two cohorts were compared using log-rank test.

A propensity score matched data set was then created in order to reduce the bias due to different patient characteristics between the two epochs (Table 1). The “Nearest match” in R (R Core Team 2020) using the MatchIt package [26] was used to match on a one-to-one ratio with calliper width of 0.06 and no replacement, matching on sex, ethnicity, high CRP on admission, Charlson comorbidity score, BMI, and pre-existing renal problem. Unpaired cases were excluded from further analysis. A further univariable analysis was performed using the matched data. Results are reported in Table 1.

As a sensitivity analysis a multivariable logistic regression was performed for each of the categorical outcomes and using the explanatory variables as described in the previous section. In order to understand the effect of the above explanatory variables on LOS, we considered Poisson regression and negative binomial (NB) regression models. The likelihood ratio test was performed to compare the two models and the NB was significantly better. Thus we used negative binomial regression with a log link to analyse LOS. P values of < 0.05 were considered statistically significant. In addition, as a sensitivity analysis an interrupted time series was performed to look at the change of the outcomes over time before and after the introduction of the AKI alert.

## Results

We briefly describe the whole hospital inpatient population during the period under investigation; there was a significant difference in the proportion of elective versus emergency admissions between the two periods (29.6 and 70.4% elective and emergency admissions, respectively in epoch 1 vs 27.2 and 72.8% in epoch 2, *p* < 0.001). A significant difference in ethnicity was also observed between the two epochs (78.1, 19.4, and 2.5% White, non-White, and unknown, epoch 1, versus 74.3, 20.5, and 5.3% epoch 2, *p* < 0.001). There was also a significant difference between the age and sex of the admitted patients between the two epochs with 40.3% and 41.6% of the patients admitted being over 55 years (*p* < 0.001) and 52.6 and 52.1% admitted males (*p* < 0.001) during epoch 1 and 2 respectively.

Overall, 23,661 patients with AKI were identified. 5505 (23.3%) were excluded because AKI status was identified whilst the patient was not an inpatient, a further 723 (4%) patients were excluded as they were aged under 18 years or already on a dialysis programme, leaving 17,433 patients to be analysed (8658 (49.7%) in epoch 1 and 8775 (50.3%) in epoch 2 (Fig. 3).

The baseline characteristics of the patients in the different groups are shown in Table 1, and results from a univariable analysis. There were significant differences in the first AKI stage, sex, ethnicity, BMI, CRP on admission, Charlson comorbidity score, and pre-existing renal problem. There was a significantly higher proportion patients with AKI stage 3 in epoch 1 (768 (8.9%) than in epoch 2 (440 (5%), *p* < 0.001). In contrast to the whole hospital population there were a significantly higher proportion of female patients in epoch 1 compared to epoch 2 (48.5% vs 45.1%, *p* < 0.001) and a higher proportion of White patients (82.8% vs 79.7%, *p* < 0.001). More patients had a high CRP on admission in epoch 1 than in epoch 2 (49.5% vs 47.5%, *p* < 0.001 respectively) and less patients had pre-existing renal problems (40.7% vs 45.5%, *p* < 0.001) (Table 1).

Following the propensity matching, 7783 patients from epoch 1 were paired with 7783 from epoch 2. All baseline demographics were no longer statistically different between the two cohorts (Table 1).

### Progression to higher AKI stage, dialysis and ICU admission

After excluding patients whose first alert was AKI stage 3, fewer patients progressed to a higher AKI stage, within 7 days of the first AKI alert (or creatinine rise), in epoch 2 compared epoch 1 (14.7% vs 17.9%, *p* < 0.001, this remained the case when analysing the matched dataset (14.8% vs 18.4%, *p* < 0.001).

The number of patients who needed dialysis, within 30 days of the first AKI alert, was 336 (3.9%) and 376 (4.3%) in epoch 1 and 2, respectively (*p* < 0.001). In the matched data, the difference was significant with more patients having dialysis in epoch2 (3.4% vs 4.4% in epoch 1 and 2, *p* < 0.001). Significantly more patients with first AKI stage 1 proceeded to having dialysis within 30 days of the first alert in epoch 2, while more patients with first AKI stage 3 had dialysis in epoch 1 in both the unmatched and matched datasets (Supplementary Table 1). Generally in the epoch 2, more patients were dialysed and more patients were dialysed at early AKI stages.

There were significantly more ICU referrals within 24 h in epoch 2 compared to epoch 1(Table 1). This is revealed in both the unmatched (84 (1%) vs 122 (1.4%), *p* = 0.0125), and matched dataset (68 (0.9%) vs 108 (1.4%), *p* = 0.004).

In the sensitivity analyses, adjusting for first AKI stage, sex, ethnicity, BMI, CRP on admission, pre-existing renal problem and Charlson scores, as detailed in Supplementary Table 1, epoch 2 were less likely to progress to a higher AKI stage (adjusted odds ratio [OR] 0.76, 95% CI 0.70–0.83, *p* = 0.001), have a higher risk of progression to dialysis within 30 days (adjusted OR 1.52, 95% CI 1.28–1.81, *p* < 0.001), and were more likely to be referred to ICU (adjusted OR 1.58, 95% CI 1.19–2.11, *p* = 0.002-Supplementary Table 1).

### Clinician behaviour – drugs, fluids and specialist referral

The percentage of patients who had drugs stopped within 12 h of the first AKI alert, was significantly lower in epoch 1 than epoch 2 (228 (2.6%) vs 324 (3.7%), *p* < 0.001). The matched dataset confirmed this with 214 (2.8%) and 281 (3.6%) patients having their drugs stopped in epoch 1 and 2, respectively (*p* = 0.0026).

The percentage of patients who had their fluids altered was approximately 17% across both epochs in both the unmatched and the matched datasets (Table 1) with no significant difference observed in the univariable analysis on the unmatched (*p* = 0.9596) or the matched data (*p* = 0.5289).

Renal referrals significantly increased after the introduction of the alert in the unmatched data (632 (7.3%) and 721 (8.2%), *p* = 0.0255) but the increase did not appear significant in the matched subset (558 (7.2%) and 621 (8%), *p* = 0.0604).

The sensitivity analyses supported these findings, in epoch2, more nephrotoxic drugs were stopped (adjusted OR 1.38, 95% CI 1.11–1.57, *p* = 0.002), there appeared no significant difference in fluid alteration (*p* = 0.748), although the chance of having a renal referrals in epoch 2 was significantly higher than in epoch 1 (*p* = 0.003) (Supplementary Table 2).

### Outcomes – death, LOS and readmission

Death during admission was slightly higher in epoch 1 (1337 (15.2%)) than in epoch 2 (1262 (14.6%)) (*p* = 0.0518). After matching death within admission was significantly lower in epoch 2 (1203 (15.5%) and 1103 (14.2%) epoch 1 and 2, respectively, *p* = 0.0255).

**Fig. 4:**
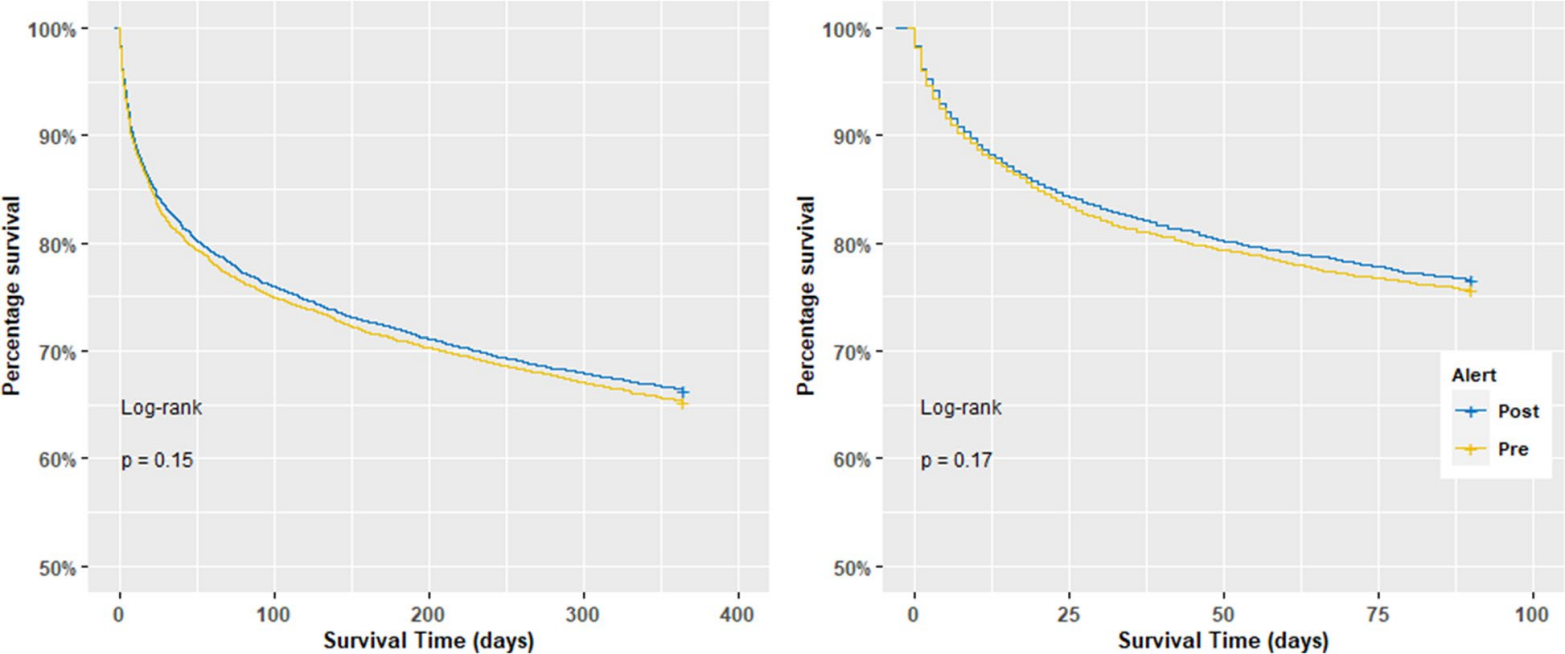
Propensity matched Kaplan–Meier curves; 1-year survival after the first alert (**A**), 90-days survival after the first alert (**B**) in the pre (yellow) and post (blue) cohorts. + shows the censored patients (figure reproduced in black and white in printed version; coloured version available online in the supplementary material)

The number of deaths within 90 days after the first AKI alert was 2114 (24.4%) and 2061 (23.5%) in epoch 1 and 2, respectively (*p* = 0.156). After propensity matching there appeared no significant difference in death rates between the epochs (1900 (24.4%) vs 1831 (23.5%), *p* = 0.2017). This remained the case when looking at time to event and there appeared no difference in Kaplan Meier curve for both epochs (Fig. 4B) (*p* = 0.170).

Similarly, although the percentage of deaths within one year of the first AKI alert was more in the epoch 1 in comparison to epoch 2, this difference was not significant (unmatched: 2990 (34.5%) vs 2934 (33.4%), *p* = 0.13, matched: 2698 (34.7%) vs 2618 (33.6%), *p* = 0.1818). The propensity matched 1-year survival curve (Fig. 4A) was also not significantly different between the two epochs (*p* = 0.149).

There was a significant difference in the median LOS between the epochs in the unmatched data (15 (IQR 7–29) vs 14 (IQR 7–28), p = 0.009) and a small but non-significant difference in the matched dataset (16 (IQR 8–30) vs 15 (IQR 8–28), *p* = 0.088).

The number of patients who had an emergency readmission within 30 days of the first alert, after excluding the patients who died on admission, was 1207 (16.5%) and 1103 (14.7%) in epoch 1 and 2, respectively (*p* = 0.003). The difference between epoch 1 and 2 was also significant after the propensity matching (1069 (16.3%) vs 968 (14.5%), *p* = 0.006).

These findings were confirmed in the sensitivity analysis; there appeared no significant difference between the epochs for patient death after discharge (*p* = 0.201 for 90 days deaths and *p* = 0.162 for death within a year). Patients in epoch 2 were less likely to die during admission (adjusted OR 0.91, 95% CI 0.83–0.99, *p* = 0.03) and had a significantly decreased chance of having an emergency readmission (adjusted OR 0.87, 95% CI 0.79–0.95, *p* = 0.003) (Supplementary Table 3). Patients in epoch 2 had a shorter stay in hospital (incidence rate ratio [IRR] = 0.94, 95% CI 0.92–0.96, *p* < 0.001) (Supplementary Table 4).

The interrupted time series analysis confirmed the results. There was an obvious step-decrease in the percentage of patients who progressed to a higher AKI after the introduction of the alert in addition to a decrease in gradient. The percentage of ICU admissions showed a step increase after the introduction of the alert then remained fairly stable. A drop in level of percentage of readmissions after the introduction of the alert was also observed. The time series of some of the different outcomes under study are shown in Supplementary Fig. 2.

## Discussion

This study demonstrates that the introduction of AKI alerts in our hospital has lowered the progression of AKI; thus likely improving long term survival [27]. This substantiates previous findings [18, 28]. Emergency readmission has also fallen after introduction of the alerts. This may be influenced by the fact that nephrotoxic drugs were stopped post alert, which has been shown in previous studies [17]. This is an example of where simple things, if done well, can alter clinician behaviour and subsequent patient outcomes. It is likely that clinicians already know how to manage AKI and alerting is merely a ‘nudge’. Even though this may only make a small change we were surprised that there was such a substantial effect on patient outcomes.

The study looked at short term (12 hourly) changes in drugs and fluid administration. We detected an increase in avoiding drugs that may interfere with kidney function, but no change in the fluid administration was observed. Wilson et al. [13] showed no difference in drug administration or fluid therapy. A time series study in an ICU setting [12], showed that the e-alerts were associated with an increase in the proportion of patients receiving fluids, diuretics and vasopressors. However, fluids are both over and under prescribed in AKI [29]. Dialysis was increased after introduction of alerts, and was earlier, as evidence by more patients at AKI stage 1 having dialysis. This presumably reflects early detection, and earlier intervention.

AKI alerts slightly increase referrals to nephrology, as seen elsewhere [13]. This has implications for renal service resource modelling. There was also a significant increase in the ICU referral and a small decrease in LOS. There was no significant influence on 90 day and 1 year survival which is substantiated by previous studies [12, 13, 19, 30] and refuted by others [31]. The population continues to age, and co-morbidities in the population continue to increase, but this is unlikely to be influential on the study as we adjusted for these in the analysis. Significant reduction in death during admission and the small decrease in LOS after alert introduction may reflect improving processes of care.

In addition to introduction of alerts in the EHR, other changes may have influenced improved patient outcomes. Guidance for AKI management was published and accessible from the Trust’s computers. The AKI alert informed doctors that this was available and directed them to help pages. It is possible therefore that overall management of the patient condition was influenced by this guidance. In addition to the interruptive AKI alert itself, there is also CDS at the point of prescribing warning if patients have recorded renal problems or deranged kidney function. This pre-dated the existence of the alerts but may have helped with ‘nudges’ after their introduction. Finally, an AKI nurse was employed in our institution subsequent to the introduction of alerts. This clinician receives all AKI 3 alerts and remotely reviews patient records, attending, and providing advice when appropriate.

Across the UK, there have been varying methods of implementing AKI alerts and the findings from the e-alert studies have been mixed. The algorithm is written into the laboratory systems and issued to organisations as a ‘result’, that is AKI Stage 1, 2 or 3. This is a UK wide initiative with a standardised algorithm. Some alerts have unsuccessfully attempted to include urine output [12]. Institutions disseminate this information in a variety of ways—via text messages to a dedicated mobile [12, 13]; stand-alone alerts [32], emails [13, 15], or in our case, and similar to another study [10], by full integration into the EHR. This may explain the difference between the findings in our study, to those in a meta-analysis [19], where no overall effect was found on outcomes (AKI progression, mortality or dialysis). This study did however note changes in doctors’ behaviour with alterations in nephrotoxic drug adjustments, fluid prescriptions and renal review.

It is highly likely that the methods by which clinicians receive alerts is influential on subsequent behaviours. We know that ‘right time, right person, right place’ facilitates changes in patients’ management [19, 33, 34]. In an integrated EHR where clinicians can see renal function changes over time, with current drugs and fluids, it is easier for doctors to act on the alert immediately, reviewing and changing medication and sending electronic referrals to renal services if needed. If the alert is via a mobile telephone, and the clinician is remote from the patient, the drug chart or the previous results, reaction may be less consistent. Training and resource are important issues and may hamper success of software [35]. We are aware of the dangers of alert fatigue [36, 37] and are careful to avoid over alerting.

There are several limitations to this study. First, as discussed above, urine output is not included. In hospitals, urine output is not uniformly well measured and therefore in practice is not helpful for screening. Urine output is not included in the national AKI algorithm. Second, since this is a retrospective study, there is an unbalanced baseline characteristics between the two cohorts. However, this was accounted for by performing the propensity score matching, before conducting the statistical analysis, to create a sample in which the two groups are balanced on baseline covariates (Table 1). The study is single-centre.

In conclusion this study supports the provision of AKI alerting; we would advocate that this should be part of a fully integrated Electronic Health Record to have a significant impact on patient safety given the complexity of the disease and its conditions that are being detected. Like checklists, alerts used well can have widespread objective changes, by subtlety altering clinician behaviour and positively changing patients’ outcomes.

## Supplementary Information

**Additional file 1.**

## Data Availability

The extraction and analysis of data for the current study was done in-line with organisational policies; it is not publically available. An anonymised participant level dataset may be made available upon receipt of an application and necessary data sharing documentation being completed, please contact the corresponding author in the first instance.
