# Supplementary material for: Does acute kidney injury alerting improve patient outcomes?
Source: BMC Nephrol. 2023 Jan 17;24:14. doi: 10.1186/s12882-022-03031-y (PMC9843843; doi:10.1186/s12882-022-03031-y)
Supplement: Supplementary file 1 — Additional file 1. [file 12882_2022_3031_MOESM1_ESM.docx]

**Supplementary Materials:**

1. **Investigated drugs and fluids:**

- ACEi/ARB: Captopril, Enalapril Maleate, Fosinopril, Imidapril, Innozide 20mg/12.5mg, Lisinopril 10mg / Hydrochlorothiazide 12.5mg, Lisinopril 20mg / Hydrochlorothiazide 12.5mg, Lisinopril, Moexipril, Perindopril, Quinapril, Ramipril, Trandolapril, Candesartan cilexetil, Entresto (sacubitril/valsartan), Eprosartan, Irbesartan with 12.5mg hydrochlorothiazide, Irbesartan, Losartan Potassium, Losartan 100mg with 12.5mg hydrochlorothiazide, Losartan 100mg with 25mg hydrochlorothiazide, Losartan 50mg with 12.5mg hydrochlorothiazide, Olmesartan medoxomil, Telmisartan 80mg with 12.5mg hydrochlorothiazide, Telmisartan 40mg with 12.5mg hydrochlorothiazide, Telmisartan, Valsartan
- Frusemide (including Furosemidein)
- Gentamycin
- Amphotericin
- NSAID: Aceclofenac, Acemetacin, Arthrotec 50, Arthrotec 75, Azapropazone, Celecoxib, Dexketoprofen, Diclofenac, Diclofenac SR, Diflunisal, Etodolac, Etodolac SR, Etoricoxib, Fenbufen, Fenoprofen, Flurbiprofen, Ibuprofen, Indometacin, Ketoprofen, Ketorolac, Mefenamic Acid, Meloxicam, Nabumetone,Naproxen, Parecoxib, Piroxicam, Rofecoxib, Sulindac, Tenoxicam, Tiaprofenic Acid, Valdecoxib
- **Fluids:** Dexsal, Gelofusine, Glucose, Sodium Chloride, Hartmanns, TPN, Sodium bicarbonate, FF HD

1. **KIDIGO guidelines:**

Acute Kidney injury (AKI): Defined according to changes in serum creatinine and described as stage 1, 2, and 3.

**Stages of AKI based on KDIGO classification:**

**Stage 1:** Increase in SCr ≥ 0.3 mg/dL (in 48 hours) or 1.5 to 1.9 multiplied by baseline (in 7 days)
**Stage 2:** 2.0 to 2.9 multiplied by baseline SCr; **Stage 3:** 3.0 or more multiplied by baseline; increase in SCr ≥ 4.0 mg/dL; or beginning of renal replacement therapy regardless of a previous KDIGO stage.


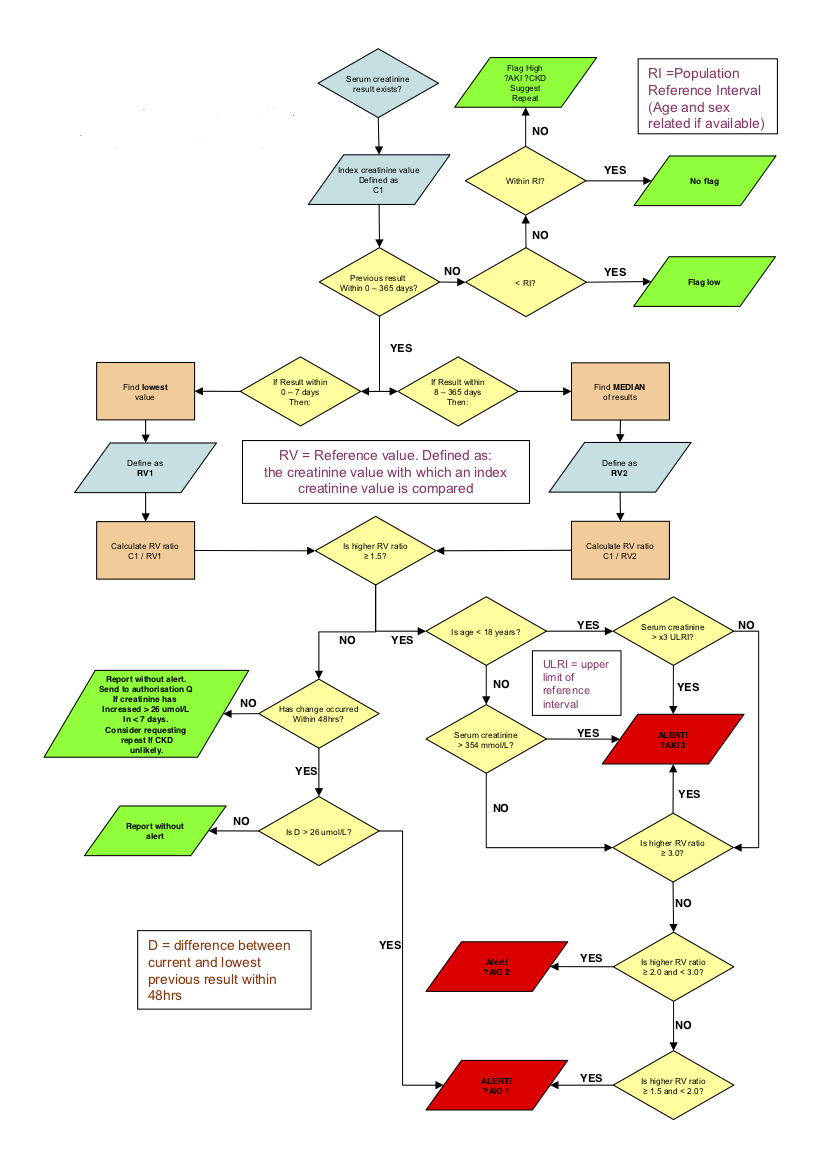


**Figure S1: Algorithm for generating E-alerts for AKI based on serum creatinine changes with time**

| **Table S1: Adjusted OR for the patient outcomes** | | | |  |  |  |  |  |  |  |  |  |
| --- | --- | --- | --- | --- | --- | --- | --- | --- | --- | --- | --- | --- |
|  |  | **Progression to higher AKI stage** | |  |  | **Dialysis after** |  |  |  | **Referred to ICU** |  |  |
|  |  | OR | 95% CI | *p* value |  | OR | 95% CI | *p* value |  | OR | 95% CI | *p* value |
| **Age** |  |  |  |  |  |  |  |  |  |  |  |  |
| 18-34 |  | 1 |  |  |  | 1 |  |  |  | 1 |  |  |
| 35-44 |  | 1.16 | 0.92 – 1.45 | 0.21 |  | 1.24 | 0.82 – 1.88 | 0.319 |  | 1.03 | 0.49 – 2.14 | 0.938 |
| 45-54 |  | 1.03 | 0.84 – 1.26 | 0.788 |  | 1.1 | 0.76 – 1.62 | 0.606 |  | 1.29 | 0.71 – 2.47 | 0.421 |
| 55-64 |  | 1.14 | 0.94 – 1.38 | 0.193 |  | 1.45 | 1.02 – 2.09 | 0.04* |  | 1.4 | 0.79 – 2.62 | 0.274 |
| 65-74 |  | 1.06 | 0.88 – 1.28 | 0.547 |  | 1.06 | 0.75 – 1.54 | 0.735 |  | 1.07 | 0.60 – 2.02 | 0.827 |
| >75 |  | 0.74 | 0.62 – 0.89 | 0.002* |  | 0.54 | 0.37 – 0.78 | 0.001* |  | 0.54 | 0.30 – 1.04 | 0.056 |
|  |  |  |  |  |  |  |  |  |  |  |  |  |
| **First AKI** |  |  |  |  |  |  |  |  |  |  |  |  |
| Stage 1 |  | 1 |  |  |  | 1 |  |  |  |  |  |  |
| Stage 2 |  | 1.24 | 1.09 – 1.40 | 0.001* |  | 1.28 | 0.93 – 1.73 | 0.118 |  | 2.79 | 1.96 – 3.91 | <0.001 |
| Stage 3 |  | _ | _ | _ |  | 11.91 | 9.84 – 14.42 | <0.001* |  | 2.7 | 1.76 – 4.03 | <0.001 |
|  |  |  |  |  |  |  |  |  |  |  |  |  |
| **Gender** |  |  |  |  |  |  |  |  |  |  |  |  |
| Male |  | 1 |  |  |  | 1 |  |  |  |  |  |  |
| Female |  | 0.85 | 0.78 – 0.93 | <0.001* |  | 0.86 | 0.72 – 1.03 | 0.095 |  | 0.6 | 0.44 – 0.81 | 0.001 |
|  |  |  |  |  |  |  |  |  |  |  |  |  |
| **Ethnicity** |  |  |  |  |  |  |  |  |  |  |  |  |
| white |  | 1 |  |  |  | 1 |  |  |  |  |  |  |
| non-white |  | 1.13 | 1.01 – 1.26 | 0.033* |  | 1.8 | 1.49 – 2.17 | <0.001* |  | 1.12 | 0.78 – 1.59 | 0.517 |
| unknown |  | 0.65 | 0.46 – 0.90 | 0.012* |  | 0.49 | 0.23 – 0.94 | 0.049* |  | 0.64 | 0.16 – 1.72 | 0.451 |
|  |  |  |  |  |  |  |  |  |  |  |  |  |
| **BMI** |  |  |  |  |  |  |  |  |  |  |  |  |
| Underweight | | 1 |  |  |  | 1 |  |  |  |  |  |  |
| Normal |  | 1.60 | 1.29 – 2.02 | <0.001* |  | 1.83 | 1.03 – 3.57 | 0.056 |  | 0.99 | 0.53 – 2.06 | 0.966 |
| Overweight |  | 1.90 | 1.52 – 2.40 | <0.001* |  | 2.67 | 1.50 – 5.20 | 0.002* |  | 1.24 | 0.65 – 2.59 | 0.541 |
| Obese |  | 1.76 | 1.41 – 2.23 | <0.001* |  | 2.53 | 1.43 – 4.94 | 0.003* |  | 0.9 | 0.47 – 1.89 | 0.758 |
| Unknown |  | 1.77 | 1.33 – 2.36 | <0.001* |  | 4.21 | 2.28 – 8.45 | <0.001* |  | 1.24 | 0.54 – 2.95 | 0.619 |
|  |  |  |  |  |  |  |  |  |  |  |  |  |
| **CRP on admission** | |  |  |  |  |  |  |  |  |  |  |  |
| low |  | 1 |  |  |  | 1 |  |  |  | 1 |  |  |
| high |  | 1.08 | 0.96 – 1.21 | 0.18 |  | 0.7 | 0.55 – 0.89 | 0.004* |  | 1.71 | 1.15 – 2.62 | 0.01 |
| Unknown |  | 1.56 | 1.37 – 1.78 | <0.001* |  | 2.56 | 2.00 – 3.29 | <0.001* |  | 1.38 | 0.84 – 2.26 | 0.204 |
|  |  |  |  |  |  |  |  |  |  |  |  |  |
| **Pre existing renal problem** | |  |  |  |  |  |  |  |  |  |  |  |
| 0 |  | 1 |  |  |  | 1 |  |  |  |  |  |  |
| 1 |  | 1.33 | 1.21 – 1.46 | <0.001* |  | 6.44 | 4.94 – 8.50 | <0.001* |  | 1.19 | 0.86 – 1.64 | 0.296 |
| Unknown |  | 0.96 | 0.83 – 1.12 | 0.62 |  | 2.93 | 2.12 – 4.08 | <0.001* |  | 1.24 | 0.73 – 2.09 | 0.424 |
|  |  |  |  |  |  |  |  |  |  |  |  |  |
| **Charlson Scores** | |  |  |  |  |  |  |  |  |  |  |  |
| 0 |  | 1 |  |  |  | 1 |  |  |  | 1 |  |  |
| 1-4 |  | 1.11 | 0.96 – 1.28 | 0.162 |  | 1.1 | 0.85 – 1.41 | 0.465 |  | 0.89 | 0.57 – 1.35 | 0.591 |
| ≥5 |  | 1.48 | 1.34 – 1.63 | <0.001* |  | 0.96 | 0.80 – 1.16 | 0.692 |  | 0.83 | 0.61 – 1.14 | 0.246 |
|  |  |  |  |  |  |  |  |  |  |  |  |  |
| **Cohort** |  |  |  |  |  |  |  |  |  |  |  |  |
| pre |  | 1 |  |  |  | 1 |  |  |  |  |  |  |
| post |  | 0.76 | 0.70 – 0.83 | <0.001* |  | 1.52 | 1.28 – 1.81 | <0.001* |  | 1.58 | 1.19 – 2.11 | 0.002 |

Multivariable logistic regression analysis adjusted for age, sex, ethnicity, BMI, CRP on admission, pre-existing renal problem, and Charlson comorbidity score.

| **Table S2: Adjusted OR for patient care process** | | | |  |  |  |  |  |  |  |  |  |
| --- | --- | --- | --- | --- | --- | --- | --- | --- | --- | --- | --- | --- |
|  |  | **Drugs stopped** |  |  |  | **Fluids altered** |  |  |  | **Referred to renal services** |  |  |
|  |  | OR | 95% CI | *p* value |  | OR | 95% CI | *p* value |  | OR | 95% CI | *p* value |
| **Age** |  |  |  |  |  |  |  |  |  |  |  |  |
| 18-34 |  | 1 |  |  |  | 1 |  |  |  | 1 |  |  |
| 35-44 |  | 0.68 | 0.35 – 1.29 | 0.246 |  | 0.98 | 0.80 – 1.21 | 0.882 |  | 1.18 | 0.85 – 1.64 | 0.321 |
| 45-54 |  | 1.15 | 0.71 – 1.91 | 0.583 |  | 1.01 | 0.85 – 1.21 | 0.884 |  | 1.04 | 0.78 – 1.41 | 0.791 |
| 55-64 |  | 1.21 | 0.77 – 1.98 | 0.424 |  | 1.01 | 0.85 – 1.20 | 0.909 |  | 1.23 | 0.93 – 1.64 | 0.149 |
| 65-74 |  | 1.6 | 1.03 – 2.57 | 0.043* |  | 0.95 | 0.80 – 1.13 | 0.559 |  | 1.14 | 0.87 – 1.51 | 0.361 |
| >75 |  | 2.16 | 1.42 – 3.43 | 0.001* |  | 0.75 | 0.64 – 0.89 | 0.001* |  | 0.94 | 0.72 – 1.24 | 0.635 |
|  |  |  |  |  |  |  |  |  |  |  |  |  |
| **First AKI** |  |  |  |  |  |  |  |  |  |  |  |  |
| Stage 1 |  | 1 |  |  |  | 1 |  |  |  | 1 |  |  |
| Stage 2 |  | 0.92 | 0.68 – 1.22 | 0.577 |  | 1.23 | 1.08 – 1.39 | 0.001* |  | 2.16 | 1.84 – 2.53 | <0.001* |
| Stage 3 |  | 0.57 | 0.36 – 0.87 | 0.013* |  | 0.95 | 0.80 – 1.12 | 0.54 |  | 3.34 | 2.83 – 3.93 | <0.001* |
|  |  |  |  |  |  |  |  |  |  |  |  |  |
|  |  |  |  |  |  |  |  |  |  |  |  |  |
| **Gender** |  |  |  |  |  |  |  |  |  |  |  |  |
| Male |  | 1 |  |  |  | 1 |  |  |  | 1 |  |  |
| Female |  | 0.8 | 0.67 – 0.96 | 0.014* |  | 0.83 | 0.76 – 0.90 | <0.001* |  | 0.77 | 0.68 – 0.87 | <0.001* |
|  |  |  |  |  |  |  |  |  |  |  |  |  |
| **Ethnicity** |  |  |  |  |  |  |  |  |  |  |  |  |
| white |  | 1 |  |  |  | 1 |  |  |  |  |  |  |
| non-white |  | 1.38 | 1.10 – 1.71 | 0.004* |  | 1.12 | 1.01 – 1.24 | 0.036* |  | 1.22 | 1.05 – 1.40 | 0.008* |
| unknown |  | 0.85 | 0.42 – 1.53 | 0.613 |  | 1.1 | 0.84 – 1.42 | 0.494 |  | 0.77 | 0.48 – 1.17 | 0.246 |
|  |  |  |  |  |  |  |  |  |  |  |  |  |
| **BMI** |  |  |  |  |  |  |  |  |  |  |  |  |
| Underweight | | 1 |  |  |  | 1 |  |  |  | 1 |  |  |
| Normal |  | 1 | 0.67 – 1.55 | 0.998 |  | 1.12 | 0.93 – 1.35 | 0.245 |  | 1.51 | 1.09 – 2.13 | 0.016* |
| Overweight |  | 1.72 | 1.16 – 2.67 | 0.01* |  | 1.15 | 0.95 – 1.39 | 0.151 |  | 2 | 1.45 – 2.83 | <0.001* |
| Obese |  | 1.21 | 0.81 – 1.88 | 0.364 |  | 1.2 | 1.00 – 1.45 | 0.056 |  | 1.75 | 1.27 – 2.48 | 0.001* |
| Unknown |  | 0.63 | 0.32 – 1.17 | 0.152 |  | 0.97 | 0.75 – 1.25 | 0.799 |  | 1.56 | 1.05 – 2.34 | 0.03* |
|  |  |  |  |  |  |  |  |  |  |  |  |  |
| **CRP on admission** | |  |  |  |  |  |  |  |  |  |  |  |
| low |  | 1 |  |  |  | 1 |  |  |  | 1 |  |  |
| high |  | 0.99 | 0.79 – 1.24 | 0.931 |  | 0.92 | 0.83 – 1.02 | 0.111 |  | 1.13 | 0.97 – 1.32 | 0.125 |
| Unknown |  | 1.16 | 0.88 – 1.52 | 0.281 |  | 1.4 | 1.24 – 1.58 | <0.001* |  | 1.33 | 1.11 – 1.60 | 0.002* |
|  |  |  |  |  |  |  |  |  |  |  |  |  |
| **Pre existing renal problem** | |  |  |  |  |  |  |  |  |  |  |  |
| 0 |  | 1 |  |  |  | 1 |  |  |  | 1 |  |  |
| 1 |  | 1.02 | 0.84 – 1.24 | 0.829 |  | 0.88 | 0.80 – 0.96 | 0.005* |  | 2.52 | 2.19 – 2.90 | <0.001* |
| Unknown |  | 0.91 | 0.65 – 1.27 | 0.602 |  | 0.87 | 0.76 – 1.01 | 0.06 |  | 1.33 | 1.05 – 1.67 | 0.015* |
|  |  |  |  |  |  |  |  |  |  |  |  |  |
| **Charlson scores** | |  |  |  |  |  |  |  |  |  |  |  |
| 0 |  | 1 |  |  |  | 1 |  |  |  | 1 |  |  |
| 1-4 |  | 1.21 | 0.89 – 1.62 | 0.212 |  | 1.26 | 1.11 – 1.43 | <0.001* |  | 1.16 | 0.96 – 1.40 | 0.126 |
| ≥5 |  | 1.47 | 1.19 – 1.82 | 0.001* |  | 1.33 | 1.21 – 1.46 | <0.001* |  | 1.4 | 1.22 – 1.60 | <0.001* |
|  |  |  |  |  |  |  |  |  |  |  |  |  |
| **Cohort** |  |  |  |  |  |  |  |  |  |  |  |  |
| pre |  |  |  |  |  |  |  |  |  | 1 |  |  |
| post |  | 1.38 | 1.11 – 1.57 | 0.002* |  | 0.99 | 0.91 – 1.07 | 0.748 |  | 1.19 | 1.06 – 1.34 | 0.003* |

Multivariable logistic regression analysis. Adjusted for age, sex, ethnicity, BMI, CRP on admission, pre-existing renal problem, and Charlson comorbidity score

| **Table S3: Adjusted OR for the patient outcomes** | | | |  |  |  |  |  |  |  |  |  |  |  |  |  |
| --- | --- | --- | --- | --- | --- | --- | --- | --- | --- | --- | --- | --- | --- | --- | --- | --- |
|  |  | **Death on admission** | |  |  | **Death within 90 days** | |  |  | **Death within a year** | |  |  | **Emergency readmission within 30 days** | | |
|  |  | OR | 95% CI | *p* value |  | OR | 95% CI | *p* value |  | OR | 95% CI | *p* value |  | OR | 95% CI | *p* value |
| **Age** |  |  |  |  |  |  |  |  |  |  |  |  |  |  |  |  |
| 18-34 |  | 1 |  |  |  | 1 |  |  |  |  |  |  |  | 1 |  |  |
| 35-44 |  | 0.94 | 0.69 – 1.28 | 0.69 |  | 1.35 | 1.03 – 1.78 | 0.029* |  | 1.5 | 1.19 – 1.89 | 0.001* |  | 1 | 0.79 – 1.26 | 0.999 |
| 45-54 |  | 1.15 | 0.88 – 1.50 | 0.306 |  | 1.75 | 1.39 – 2.22 | <0.001* |  | 1.91 | 1.57 – 2.34 | <0.001* |  | 1.02 | 0.84 – 1.26 | 0.815 |
| 55-64 |  | 1.41 | 1.11 – 1.81 | 0.006* |  | 2.19 | 1.76 – 2.75 | <0.001* |  | 2.36 | 1.95 – 2.86 | <0.001* |  | 0.91 | 0.75 – 1.11 | 0.363 |
| 65-74 |  | 1.8 | 1.42 – 2.29 | <0.001* |  | 2.68 | 2.16 – 3.35 | <0.001* |  | 2.88 | 2.39 – 3.48 | <0.001* |  | 0.95 | 0.79 – 1.16 | 0.636 |
| >75 |  | 2.8 | 2.24 – 3.54 | <0.001* |  | 4.49 | 3.65 – 5.57 | <0.001* |  | 5.01 | 4.19 – 6.03 | <0.001* |  | 0.79 | 0.66 – 0.96 | 0.018* |
|  |  |  |  |  |  |  |  |  |  |  |  |  |  |  |  |  |
| **First AKI** |  |  |  |  |  |  |  |  |  |  |  |  |  |  |  |  |
| Stage 1 |  | 1 |  |  |  | 1 |  |  |  | 1 |  |  |  | 1 |  |  |
| Stage 2 |  | 1.64 | 1.44 – 1.86 | <0.001* |  | 1.7 | 1.52 – 1.90 | <0.001* |  | 1.52 | 1.36 – 1.69 | <0.001* |  | 0.96 | 0.82 – 1.11 | 0.563 |
| Stage 3 |  | 1.32 | 1.12 – 1.55 | 0.001* |  | 1.38 | 1.20 – 1.59 | <0.001* |  | 1.25 | 1.09 – 1.43 | 0.001* |  | 1.22 | 1.02 – 1.45 | 0.025* |
|  |  |  |  |  |  |  |  |  |  |  |  |  |  |  |  |  |
| **Sex** |  |  |  |  |  |  |  |  |  |  |  |  |  |  |  |  |
| Male |  | 1 |  |  |  | 1 |  |  |  | 1 |  |  |  | 1 |  |  |
| Female |  | 0.88 | 0.81 – 0.97 | 0.006* |  | 0.9 | 0.83 – 0.97 | 0.005* |  | 0.9 | 0.84 – 0.96 | 0.002* |  | 1.02 | 0.93 – 1.12 | 0.651 |
|  |  |  |  |  |  |  |  |  |  |  |  |  |  |  |  |  |
| **Ethnicity** |  |  |  |  |  |  |  |  |  |  |  |  |  |  |  |  |
| white |  | 1 |  |  |  | 1 |  |  |  | 1 |  |  |  | 1 |  |  |
| non-white |  | 1.12 | 0.99 – 1.26 | 0.067 |  | 0.95 | 0.86 – 1.06 | 0.372 |  | 0.88 | 0.80 – 0.96 | 0.007* |  | 1.07 | 0.95 – 1.20 | 0.269 |
| unknown |  | 1.41 | 1.04 – 1.88 | 0.024* |  | 1.22 | 0.93 – 1.58 | 0.15 |  | 0.84 | 0.64 – 1.08 | 0.171 |  | 0.59 | 0.38 – 0.87 | 0.012* |
|  |  |  |  |  |  |  |  |  |  |  |  |  |  |  |  |  |
| **BMI** |  |  |  |  |  |  |  |  |  |  |  |  |  |  |  |  |
| Underweight (≤18.5) | | 1 |  |  |  | 1 |  |  |  | 1 |  |  |  | 1 |  |  |
| Normal(18.5-24.9) | | 0.73 | 0.62 – 0.86 | <0.001* |  | 0.59 | 0.51 – 0.68 | <0.001* |  | 0.56 | 0.49 – 0.65 | <0.001* |  | 1.07 | 0.87 – 1.32 | 0.522 |
| Overweight (25-30) | | 0.49 | 0.41 – 0.59 | <0.001* |  | 0.35 | 0.30 – 0.41 | <0.001* |  | 0.31 | 0.27 – 0.36 | <0.001* |  | 1.13 | 0.92 – 1.41 | 0.257 |
| Obese (>30) | | 0.56 | 0.47 – 0.67 | <0.001* |  | 0.41 | 0.35 – 0.48 | <0.001* |  | 0.37 | 0.32 – 0.43 | <0.001* |  | 1.09 | 0.89 – 1.35 | 0.419 |
| Unknown | | 0.87 | 0.69 – 1.09 | 0.231 |  | 0.7 | 0.57 – 0.85 | <0.001* |  | 0.52 | 0.43 – 0.64 | <0.001* |  | 0.98 | 0.73 – 1.31 | 0.9 |
|  |  |  |  |  |  |  |  |  |  |  |  |  |  |  |  |  |
| **CRP on admission** | |  |  |  |  |  |  |  |  |  |  |  |  |  |  |  |
| Low (≤5 mg/L) | | 1 |  |  |  | 1 |  |  |  | 1 |  |  |  | 1 |  |  |
| High (>5 mg/L) | | 1.83 | 1.62 – 2.07 | <0.001* |  | 2.15 | 1.94 – 2.38 | <0.001* |  | 2.21 | 2.02 – 2.42 | <0.001* |  | 1.15 | 1.03 – 1.28 | 0.015* |
| Unknown | | 1.12 | 0.96 – 1.32 | 0.161 |  | 1.12 | 0.98 – 1.28 | 0.102 |  | 1.11 | 0.98 – 1.25 | 0.089 |  | 0.57 | 0.49 – 0.66 | <0.001* |
|  |  |  |  |  |  |  |  |  |  |  |  |  |  |  |  |  |
| **Pre existing renal problem** | |  |  |  |  |  |  |  |  |  |  |  |  |  |  |  |
| 0 |  | 1 |  |  |  | 1 |  |  |  | 1 |  |  |  | 1 |  |  |
| 1 |  | 1.29 | 1.17 – 1.43 | <0.001* |  | 1.24 | 1.14 – 1.35 | <0.001* |  | 1.22 | 1.13 – 1.32 | <0.001* |  | 1.13 | 1.02 – 1.25 | 0.016* |
| Unknown |  | 1.7 | 1.43 – 2.03 | <0.001* |  | 1.82 | 1.56 – 2.11 | <0.001* |  | 1.59 | 1.39 – 1.82 | <0.001* |  | 0.6 | 0.48 – 0.75 | <0.001* |
|  |  |  |  |  |  |  |  |  |  |  |  |  |  |  |  |  |
| **Charlson Scores** | |  |  |  |  |  |  |  |  |  |  |  |  |  |  |  |
| 0 |  | 1 |  |  |  | 1 |  |  |  | 1 |  |  |  | 1 |  |  |
| 1-4 |  | 0.93 | 0.79 – 1.09 | 0.351 |  | 0.91 | 0.79 – 1.03 | 0.147 |  | 0.96 | 0.85 – 1.08 | 0.458 |  | 1.38 | 1.20 – 1.58 | <0.001* |
| ≥5 |  | 1.89 | 1.70 – 2.10 | <0.001* |  | 2.08 | 1.90 – 2.28 | <0.001* |  | 2.24 | 2.07 – 2.43 | <0.001* |  | 1.33 | 1.20 – 1.48 | <0.001* |
|  |  |  |  |  |  |  |  |  |  |  |  |  |  |  |  |  |
| **Cohort** |  |  |  |  |  |  |  |  |  |  |  |  |  |  |  |  |
| pre |  |  |  |  |  |  |  |  |  | 1 |  |  |  | 1 |  |  |
| post |  | 0.91 | 0.83 – 0.99 | 0.03* |  | 0.95 | 0.88 – 1.03 | 0.201 |  | 0.95 | 0.88 – 1.02 | 0.162 |  | 0.87 | 0.79 – 0.95 | 0.003* |

Multivariable logistic regression analysis. Adjusted for age, sex, ethnicity, BMI, CRP on admission, pre-existing renal problem, and Charlson comorbidity score

| **Table S4: Adjusted IRR** | |  |  |
| --- | --- | --- | --- |
|  | **Length of hospital stay** | | |
|  | IRR | 95% CI | *p* value |
| **Age** |  |  |  |
| 18-34 | 1 |  |  |
| 35-44 | 1.04 | 0.97 – 1.12 | 0.262 |
| 45-54 | 1 | 0.94 – 1.06 | 0.958 |
| 55-64 | 1.08 | 1.01 – 1.14 | 0.018 |
| 65-74 | 1.01 | 0.95 – 1.07 | 0.816 |
| >75 | 1.04 | 0.99 – 1.11 | 0.136 |
|  |  |  |  |
| **First AKI** |  |  |  |
| Stage 1 | 1 |  |  |
| Stage 2 | 0.97 | 0.93 – 1.02 | 0.239 |
| Stage 3 | 0.89 | 0.84 – 0.94 | <0.001 |
|  |  |  |  |
|  |  |  |  |
| **Gender** |  |  |  |
| Male | 1 |  |  |
| Female | 0.96 | 0.93 – 0.99 | 0.006 |
|  |  |  |  |
| **Ethnicity** |  |  |  |
| white | 1.00 |  |  |
| non-white | 0.97 | 0.94 – 1.01 | 0.179 |
| unknown | 0.61 | 0.55 – 0.67 | <0.001 |
|  |  |  |  |
| **BMI** |  |  |  |
| Underweight | 1 |  |  |
| Normal | 0.94 | 0.89 – 1.00 | 0.043 |
| Overweight | 0.84 | 0.79 – 0.90 | <0.001 |
| Obese | 0.87 | 0.82 – 0.92 | <0.001 |
| Unknown | 0.8 | 0.74 – 0.87 | <0.001 |
|  |  |  |  |
| **CRP on admission** | |  |  |
| low | 1 |  |  |
| high | 1.1 | 1.06 – 1.14 | <0.001 |
| Unknown | 1.12 | 1.07 – 1.17 | <0.001 |
|  |  |  |  |
| **Pre-existing renal problem** | |  |  |
| 0 | 1 |  |  |
| 1 | 0.94 | 0.91 – 0.97 | <0.001 |
| Unknown | 1.26 | 1.20 – 1.33 | <0.001 |
|  |  |  |  |
| **Charlson score** |  |  |  |
| 0 | 1 |  |  |
| 1-4 | 1.09 | 1.05 – 1.14 | <0.001 |
| ≥5 | 1.2 | 1.17 – 1.24 | <0.001 |
|  |  |  |  |
| **Cohort** |  |  |  |
| pre | 1 |  |  |
| post | 0.94 | 0.92 – 0.98 | <0.001 |

**Multivariable negative binomial regression analysis. Adjusted for age, sex, ethnicity, BMI, CRP on admission, pre-existing renal problem, and Charlson comorbidity score**

| **Table S5: Dialysis patients in the pre and post cohort by First AKI stage (unmatched cohort)** | | |
| --- | --- | --- |
|  | **Cohort** |  |
|  | pre | post |
| **First AKI** |  |  |
| Stage 1 | 82 (11.5%) | 269 (37.8%) |
| Stage 2 | 28 (3.9%) | 22 (3.1%) |
| Stage 3 | 226 (31.7%) | 85 (11.9%) |
|  |  |  |
|  |  |  |
|  |  |  |
| **Table S6: Dialysis patients in the pre and post cohort by First AKI stage (matched cohort)** | | |
|  | **Cohort** |  |
|  | pre | post |
| **First AKI** |  |  |
| Stage 1 | 81 (13.5%) | 233 (38.7%) |
| Stage 2 | 26 (4.3%) | 22 (3.7%) |
| Stage 3 | 155 (25.8%) | 85 (14.1%) |


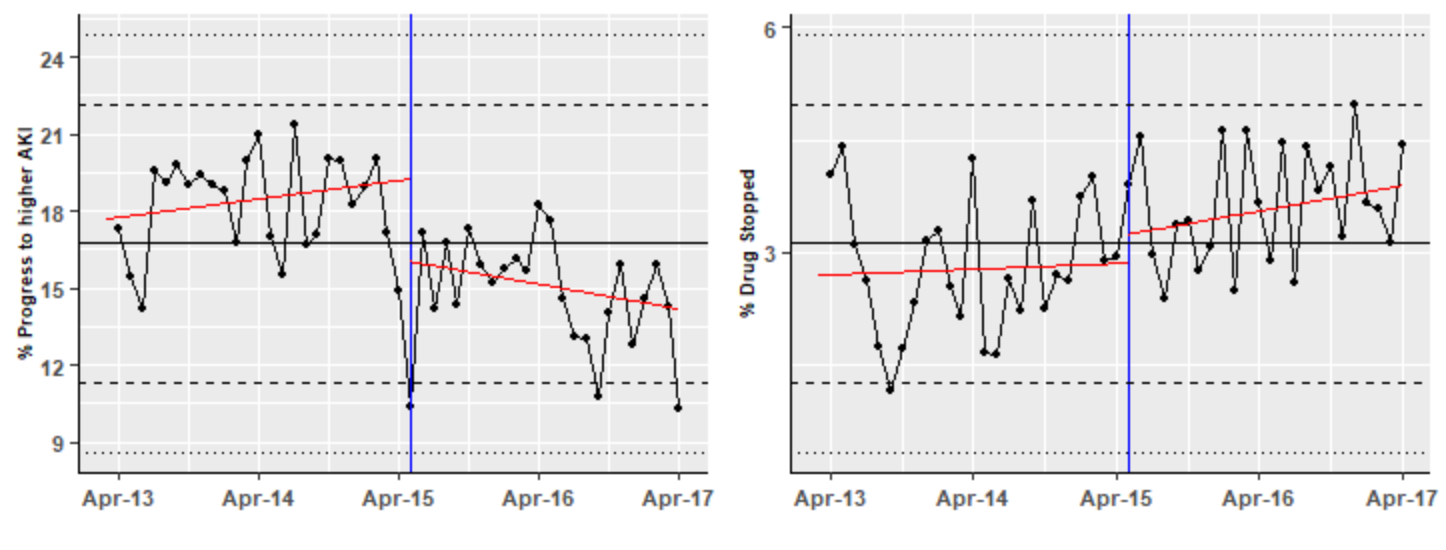


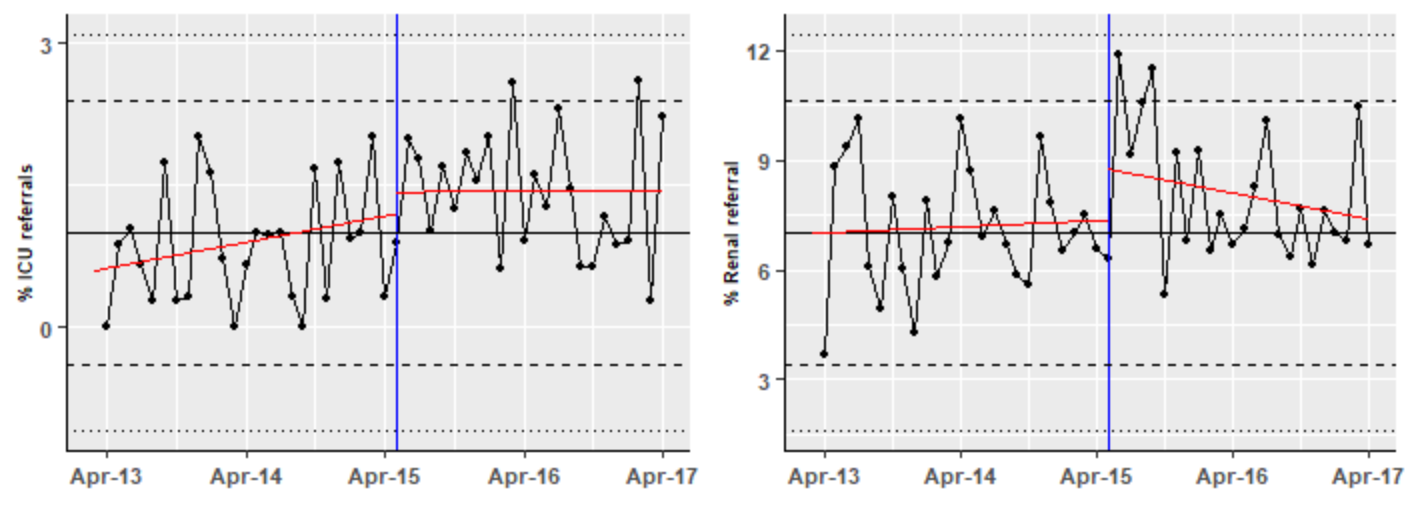


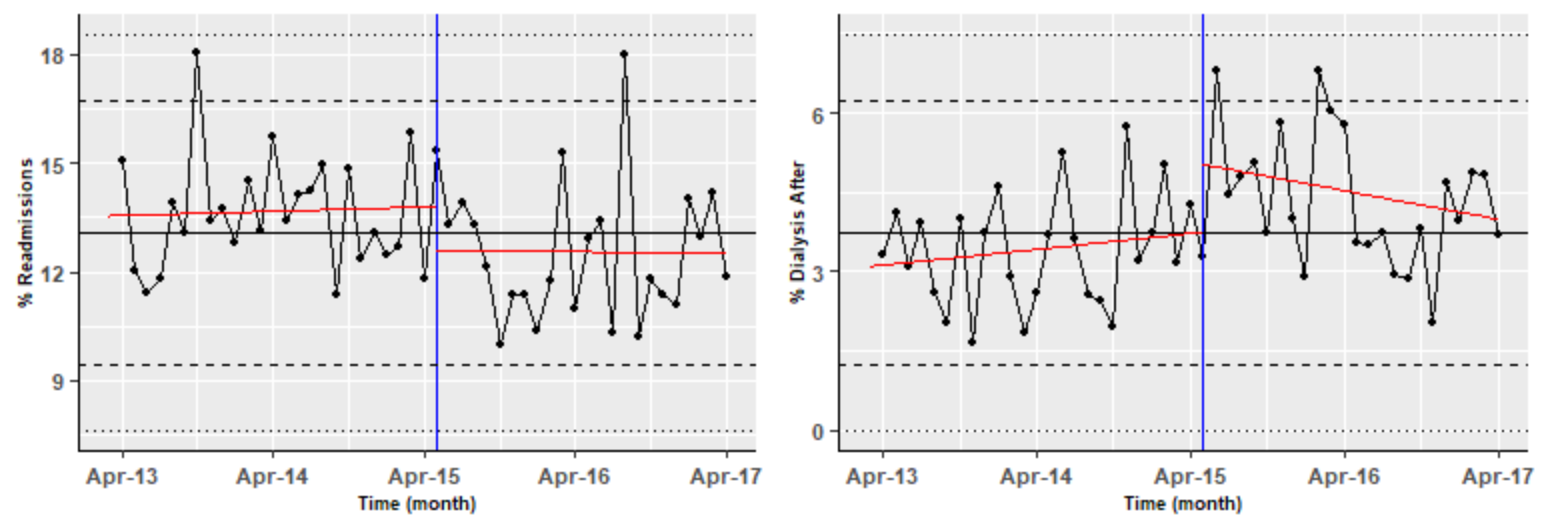


**Figure S2: Time series of outcomes (as a percentage of the number of monthly AKI alerts). Also shown the linear regression model fit (trend lines) for each segment (red line), the intervention time (blue), median of the whole data (black). Two and three standard deviation from the median (dashed and dotted black lines, respectively).**
